# *Schizosaccharomyces pombe* MutSα and MutLα Maintain Stability of Tetra-Nucleotide Repeats and Msh3 of Hepta-Nucleotide Repeats

**DOI:** 10.1534/g3.117.040816

**Published:** 2017-03-21

**Authors:** Desirée Villahermosa, Olaf Christensen, Karen Knapp, Oliver Fleck

**Affiliations:** * North West Cancer Research Institute, School of Medical Sciences, Bangor University, LL57 2UW, UK; † Strain Development LBPE38, Lonza, CH-3930 Visp, Switzerland

**Keywords:** mismatch repair, microsatellite instability, homologous recombination repair, repetitive DNA, FEN1

## Abstract

Defective mismatch repair (MMR) in humans is associated with colon cancer and instability of microsatellites, that is, DNA sequences with one or several nucleotides repeated. Key factors of eukaryotic MMR are the heterodimers MutSα (Msh2-Msh6), which recognizes base-base mismatches and unpaired nucleotides in DNA, and MutLα (Mlh1-Pms1), which facilitates downstream steps. In addition, MutSβ (Msh2-Msh3) recognizes DNA loops of various sizes, although our previous data and the data presented here suggest that Msh3 of *Schizosaccharomyces pombe* does not play a role in MMR. To test microsatellite stability in *S. pombe* and hence DNA loop repair, we have inserted tetra-, penta-, and hepta-nucleotide repeats in the *ade6* gene and determined their Ade^+^ reversion rates and spectra in wild type and various mutants. Our data indicate that loops with four unpaired nucleotides in the nascent and the template strand are the upper limit of MutSα- and MutLα-mediated MMR in *S. pombe*. Stability of hepta-nucleotide repeats requires Msh3 and Exo1 in MMR-independent processes as well as the DNA repair proteins Rad50, Rad51, and Rad2^FEN1^. Most strikingly, mutation rates in the double mutants *msh3 exo1* and *msh3 rad51* were decreased when compared to respective single mutants, indicating that Msh3 prevents error prone processes carried out by Exo1 and Rad51. We conclude that Msh3 has no obvious function in MMR in *S. pombe*, but contributes to DNA repeat stability in MMR-independent processes.

Repetitive DNA elements are widespread in genomes. They are located in centromeres, telomeres, rDNA genes, transposons, and intergenic regions (Padeken *et al.* 2015). One class of repetitive DNA comprises microsatellites, which are short tandem repeats of one to several nucleotides. Their lengths often differ between individuals of a given species, but are identical in cells of an individual organism. During DNA replication, repeat units can slip and misalign with repeats of the opposite strand. Unrecognized slippage in the template strand leads to deletions, and in the nascent strand to insertions after the next round of replication. The major pathway for maintaining repeat tract lengths of microsatellites is mismatch repair (MMR). Eukaryotic MMR is initiated either by Msh2-Msh6 (MutSα) or by Msh2-Msh3 (MutSβ) (Alani 1996; Habraken *et al.* 1996; Johnson *et al.* 1996; Marsischky *et al.* 1996). Depending on the eukaryote, some differences exist in the recognition spectrum of the two complexes (Marti *et al.* 2002). Generally, MutSα is responsible for repair of base-base mismatches and loops, and MutSβ for repair of loops. After mismatch binding, MutL heterodimers are recruited, with Mlh1-Pms1 (MutLα) (termed Mlh1-Pms2 in humans) being the major factor for MMR (Jiricny 2013). The newly synthesized strand with the erroneous nucleotide(s) is subsequently degraded either by the 5′-exonuclease activity of Exo1 or by the endonuclease activity of MutLα (Kadyrov *et al.* 2006, 2007; Smith *et al.* 2013; Goellner *et al.* 2014, 2015). The homotrimeric processivity factor PCNA binds to MutSα, MutSβ, and MutLα and coordinates correct incision of the newly synthesized strand (Flores-Rozas *et al.* 2000; Kleczkowska *et al.* 2001; Lee and Alani 2006; Iyer *et al.* 2010; Pluciennik *et al.* 2010). After excision beyond the mismatch, a replicative DNA polymerase fills the resulting gap and the remaining nick is ligated.

A defect in the human MMR genes *MSH2* and *MLH1* causes microsatellite instability and a predisposition to colon and other types of cancer (Lynch *et al.* 2009; Da Silva *et al.* 2016). Mutations in other MMR genes are rarely correlated with cancer, probably due to functional redundancy. In contrast to other types of repetitive elements, microsatellites are often situated in genes, which is a critical factor for tumor development and important for the choice of drugs for treatment of cancer patients. For example, deletions in a T_11_ repeat in intron 4 of *MRE11* causes aberrant splicing, and as a consequence, a truncated MRE11 protein (Giannini *et al.* 2002). This sensitizes cancer cells to combined treatment with camptothecin and thymidine (Rodriguez *et al.* 2008).

Recombination processes can alter tract lengths of repetitive DNA either by unequal crossover between repeats or through secondary structures formed between repeats in the same strand. The Mre11-Rad50-Nbs1 (MRN) complex has single-stranded 3′-exonuclease and endonuclease activities as well as structural functions in recombination processes (Cejka 2015). After 5′–3′ resection of DNA double-strand breaks or ends by Exo1 or other 5′-exonucleases, Rad51-dependent homologous recombination (HR) can occur by invasion of the 3′-ended single strand into a complimentary DNA molecule. Rad51-independent single-strand annealing (SSA) can also occur between two repeats and leads to deletion of the intervening sequence. SSA requires the nucleotide excision repair factors Rad1-Rad10 of *Saccharomyces cerevisiae* (XPF-ERCC1 in human) and MutSβ (Bhargava *et al.* 2016). FEN1 is a flap endonuclease with multiple roles in DNA metabolisms. FEN1 is involved in processing of Okazaki fragments during replication, in long-patch base excision repair and in other processes (Marti and Fleck 2004). *S. cerevisiae*, *rad27* (a FEN1 homolog) mutants exhibit instability of mono- and dinucleotide repeats and generate duplications of sequences flanked by repeats (Johnson *et al.* 1995; Tishkoff *et al.* 1997; Kirchner *et al.* 2000). In addition, FEN1 has been implicated in trinucleotide repeat stability (Freudenreich *et al.* 1998; Liu and Wilson 2012) and repair of large loops with up to 216 unpaired nucleotides (Sommer *et al.* 2008).

The genome of fission yeast *Schizosaccharomyces pombe* encodes the MutS homologs Msh1, Msh2, Msh3, and Msh6, the MutL homologs Mlh1 and Pms1, and the exonuclease Exo1. Based on homology with *S. cerevisiae* Msh1, *S. pombe* Msh1 likely acts in MMR of mitochondrial DNA. *S. pombe* Msh2, Msh6, Mlh1, and Pms1 are indispensable for repair of base-base mismatches and small loops with one or two nucleotides (Schär *et al.* 1997; Rudolph *et al.* 1999; Mansour *et al.* 2001; Tornier *et al.* 2001; Marti *et al.* 2003). In contrast, Msh3 seems to have no, or a minor and rather MMR-independent, function in repair of base-base mismatches and small loops. Exo1 appears to be involved in MMR of base-base mismatches but has a rather MMR-independent function in repair of small loops (Rudolph *et al.* 1998; Mansour *et al.* 2001; Marti *et al.* 2003).

In the present study, we tested stability of tetra-, penta-, and hepta-nucleotide repeats in *S. pombe*. Our aim was to analyze whether stability of such repeats is dependent on MMR, and if so, whether MutSα, MutSβ, or both are involved. In addition, we analyzed *mlh1*, and thus MutLα-deficient strains as well as *exo1* mutants.

## Materials and Methods

### General yeast genetic methods, media, and S. pombe strains

The *S. pombe* media minimal medium agar (MMA), yeast extract agar (YEA), and yeast extract liquid (YEL), and general genetic methods were used as described (Gutz *et al.* 1974). *S. pombe* strains used in this study were derived from Ru39 *h^−^ msh2*::*his3 his3-D1* (Rudolph *et al.* 1999); KK83 *smt-0 msh3*::*loxP-ura4-loxM leu1-32 ura4-D18* (D. Villahermosa, K. Knapp, and O. Fleck, unpublished data); OL937 *h^−^ mlh1*::*kanMX his3-D1 ura4-D18* (Marti *et al.* 2003); Ru42 *h^−^ exo1*::*ura4 ura4-D18* (Szankasi and Smith 1995; Rudolph *et al.* 1998); sp217 *h^−^ rad2*::*ura4 ade6-704 leu1-32 ura4-D18* (Murray *et al.* 1994); EH65 *smt-0 rad50*::*kanMX ura4-D18* (Hartsuiker *et al.* 2001), and SPAC644.14c *h^+^ rad51*::*kanMX ade6 leu1-32 ura4-D18* (Kim *et al.* 2010).

Strains KK42 *h^−^ msh2*::*hphMX ura4-D18* and KK37 *h^−^ msh6*::*hphMX ura4-D18* originated from transformations of OL2137 (*h^−^ ura4-D18*), with DNA fragments obtained by fusion PCRs. pFA6a-hphMX (Hentges *et al.* 2005) was used as template to amplify the hygromycin resistance cassette. Genomic DNA of strain RO144 (*smt-0*) was used to amplify either 450 bp of the 3′ UTR of the *msh2* locus or 500 bp of the 5′ UTR of the *msh6* locus. Primers for the *msh2* disruption were msh2_For 5′-GAGGTTTTTTATTTATCCTTTTTGAGGACTTAACTGTGGCAAGGAGTTTCTTCTCCTGTTTTATACATTTCGCGTTCGCGCTTTAGAACATTCAATCAATCGGATCCCCGGGTTAATTAA; msh2_Rev 5′-TTTCCTCGTTTTAGTAAAAAATTATTTTATTCATAAAATGCGCTTCCAAAAAACATGTACCTTGGTTGAATTCTTTCAATTAGTACCTTGCTCACATTCTGAATTCGAGCTCGTTTAAAC; msh2_For3 5′-TTGAAAGAATTCAACCAAGG; and msh2_Rev3 5′-GCTAAAACAAAATTATGCCG. Primers for the *msh6* disruption were msh6_For 5′-TATATATGTTATTTTGTGCTCTCATGTTAGCTTTGTTTACTATTAGAATGCTGCTTTTTGTAAATAACTGAACTTAGCCAAAACCAACACTTGTTCCAGTCGGATCCCCGGGTTAATTAA; msh6_Rev 5′-ATAACGTAAGTAAATGGTAAATAAAAGCAAGCTTCCGCTTGCCAGCAAACGAAAGATATTGCTTTGAATAGTCATAAAACTGATAGAGTGTTGACAGTTAGAATTCGAGCTCGTTTAAAC; msh6_For2 5′-CTCATCTTACCTAAACTCTC; and msh6_Rev2 5′-GAACAAGTGTTGGTTTTGGC.

### Construction of (GACC)_n_ repeats in the ade6 gene

In pAN-K, a pUC18 derivative, the kanamycin resistance gene was replaced by a 340-bp *Dra*III-*Hin*dIII fragment of the *ade6* gene containing a (GACC)_7_ repeat near the *Dra*III site (underlined in primer ade6-GACC7; see below). The fragment was obtained by PCR with primers ade6-GACC7 5′-TCCCACTTGGTGACCGACCGACCGACCGACCGACCGACCTTTATGTTGAAAAGTTCGTTC-3′ and ade6-H 5′-GGGCAAGCTTCAATGGTGTA-3′, and subsequent digestion with *Dra*III and *Hin*dIII. PCR was performed under standard conditions using pCG162 as template (Grimm *et al.* 1994). Plasmids with the desired insert were identified by restriction digests and DNA sequencing. In addition, a plasmid was identified in which one T in a T_3_ run immediately 3′ to the (GACC)_7_ repeat was deleted. The 1.7-kb *Xho*I-*Eco*RI fragments of the plasmids containing either (GACC)_7_ or (GACC)_7_ΔT were transformed into the *S. pombe* strain AM1 (*h^–^ ade6*::*ura4 his3-D1 ura4-D18 leu1-32*; Mansour *et al.* 2001). Correct integration was confirmed by PCR and DNA sequencing. The resulting strains were *h^–^ ade6-(GACC)_7_ his3-D1 leu1-32 ura4-D18* and *h^–^ ade6-(GACC)_7_*Δ*T his3-D1 leu1-32 ura4-D18*. A strain with a (GACC)_8_ repeat was isolated during a fluctuation test with an *h*^+^ *msh2*::*his3 ade6-(GACC)_7_* mutant.

### Construction of the ade6::ura4 strain DE1

**Figure 1:**
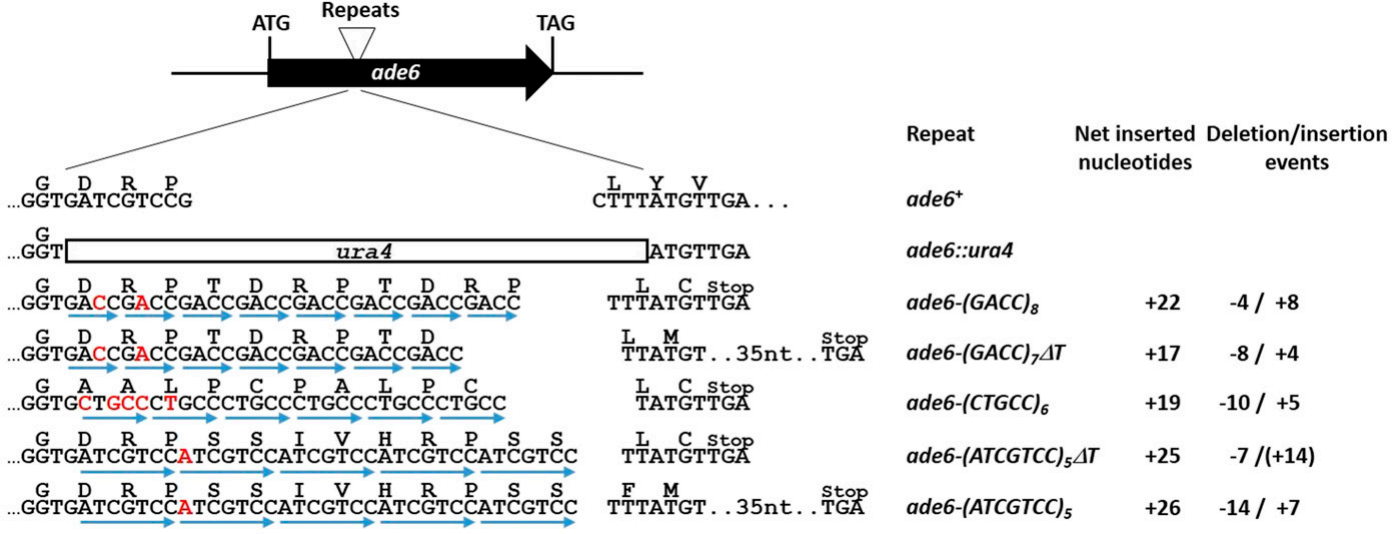
Schematic of the position and nature of the various repeats. In the *ade6*::*ura4* disruption strain, 13 nucleotides have been deleted and replaced by the *ura4* marker. This strain was transformed with DNA fragments to produce *ade6* mutants with the indicated repeats (highlighted by blue arrows). Nucleotides within the repeats that differ from the wild-type sequence are shown in red. Integration of the repeats caused frameshifts with nearby located stop codons. The numbers of net inserted nucleotides and of the major deletion/insertion events that lead to Ade^+^ reversions are given on the right. (+14) indicates that, although this event restores the open reading frame, it was not found among the 30 *ade6-(ATCGTCC)_5_*Δ*T* revertants sequenced (Table 6). In (GACC)_7_ΔT and (ATCGTCC)_5_ΔT, a T in a T_3_ stretch immediately downstream of the repeats is deleted. This T deletion allows detecting different types of deletions and insertions within respective repeats in comparison to (GACC)_8_ and (ATCGTCC)_5_, as indicated on the right.

The strain DE1 (*h^−^ ade6*::*ura4 ura4-D18*) was constructed by transformation of strain OL2137 (*h^−^ ura4-D18*) with a PCR fragment obtained with primers ade6_d_ura4_F 5′-TCCTTTTGTACTGAAAAGTAAAACATTGGCTTACGACGGTCGTGGAAATTACGTTGTTCATCAACCATCTGAGATTCCTACTGCCATCAAAGCACTTGGTagcttagctacaaatcccac and ade6_d_ura4_R 5′-GAATGGTCTCAGTTGTAGGATAAGCATAAACTTTTCCGTCTAAACTGCGTACTACCATCACTGCAATTTCCATGGAGAAAGGAACGAACTTTTCAACATagcttgtgatattgacgaaac, and pAW1 as template (Watson *et al.* 2008). Nucleotides in lowercase letters were derived from the *ura4* marker gene; nucleotides in capital letters were derived from *ade6*. In this way, 13 nucleotides (5′-GATCGTCCGCTTT) in *ade6* were deleted and replaced by *ura4* (Figure 1).

### Construction of penta- and hepta-nucleotide repeats in the ade6 gene

We subjected 100 pmol of each of two 120-nucleotide-long oligonucleotides, with ∼40 nucleotides complementary to each other at their 3′ ends, to primer extension in 50 μl reactions containing 1 U Go*Taq* polymerase, 2.5 mM MgCl_2_, 50 μM each dNTP in Colorless Go*Taq* Flexi buffer (Promega). After initial denaturation for 1 min at 94°, we applied two cycles with 30 sec at 94°, 30 sec at 45°, and 30 sec at 72°, followed by five cycles with 30 sec at 94°, 30 sec at 55°, and 30 sec at 72°. Reaction samples were transformed into strain DE1 using the method of Ito *et al.* (1983) with some modifications (D. Villahermosa, K. Knapp, and O. Fleck, unpublished data). Primers Penta2-F 5′-TAAAACATTGGCTTACGACGGTCGTGGAAATTACGTTGTTCATCAACCATCTGAGATTCCTACTGCCATCAAAGCACTTGGTGCTGCCCTGCCCTGCCCTGCCCTGCCCTGCCTATGTTG and Penta2-R 5′-GGATAAGCATAAACTTTTCCGTCTAAACTGCGTACTACCATCACTGCAATTTCCATGGAGAAAGGAACGAACTTTTCAACATAGGCAGGGCAGGGCAGGGCAGGGCAGGGCAGCACCAAG were used to construct *ade6-(CTGCC)_6_*. Primers Hepta-F 5′-ACATTGGCTTACGACGGTCGTGGAAATTACGTTGTTCATCAACCATCTGAGATTCCTACTGCCATCAAAGCACTTGGTGATCGTCCATCGTCCATCGTCCATCGTCC ATCGTCCTTTATG and Hepta-R 5′-AAGCATAAACTTTTCCGTCTAAACTGCGTACTACCATCACTGCAATTTCCATGGAGAAAGGAACGAACTTTTCAACATAAAGGACGATGGACGATGGACGATGGACGATGGACGATCACC were used for *ade6-(ATCGTCC)_5_*. Primers HeptadT-F 5′-AACATTGGCTTACGACGGTCGTGGAAATTACGTTGTTCATCAACCATCTGAGATTCCTACTGCCATCAAAGCACTTGGTGATCGTCCATCGTCCATCGTCCATCGTCCATCGTCCTTATG and HeptadT-R 5′-TAAGCATAAACTTTTCCGTCTAAACTGCGTACTACCATCACTGCAATTTCCATGGAGAAAGGAACGAACTTTTCAACATAAGGACGATGGACGATGGACGATGGACGATGGACGATCACC were used for *ade6-(ATCGTCC)_5_*Δ*T*. Here, a single T immediately downstream of the repeats has been deleted (Figure 1).

### Determination of mutation rates and spectra

Mutation rates were determined by fluctuation tests as described (Mansour *et al.* 2001). In brief, seven tubes containing 2 ml YEL were each inoculated with a single small colony and incubated at 30° until cultures were grown to stationary phase. Appropriate dilutions were plated on YEA for cell titer determination and on MMA for selection of Ade^+^ revertants. Colonies were counted after 5 d of growth at 30°, except for strains with *rad50* or *rad51* background, where colonies were counted after 6 d to compensate for their slow growth. Reversion rates were calculated from at least three independent fluctuation tests. Statistical significance was calculated with a two-tailed Student’s *t*-test.

**Figure 2:**
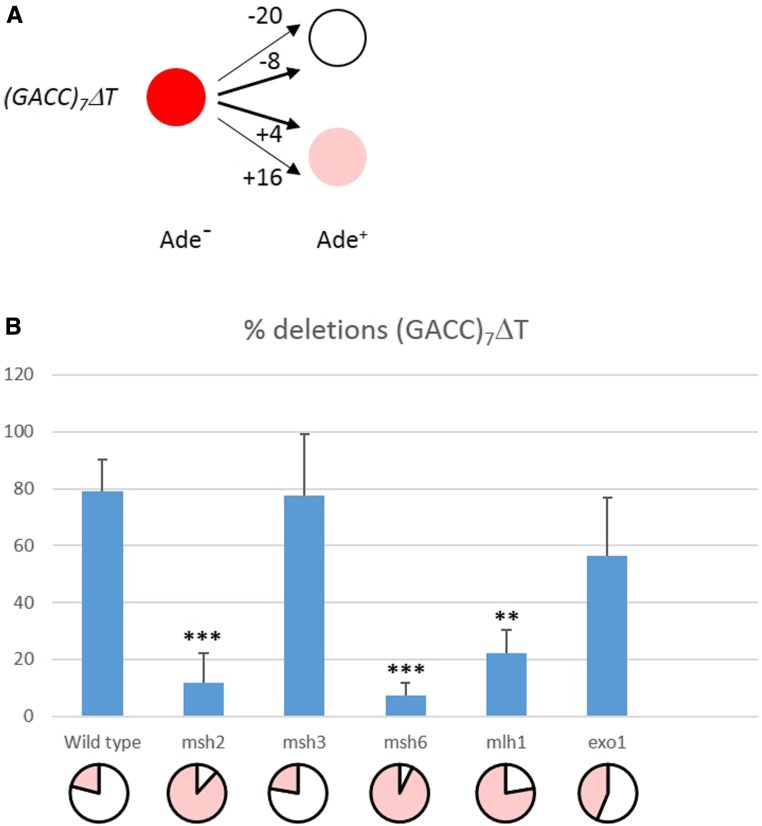
Distribution of deletions and insertions in the *ade6-(GACC)_7_*Δ*T* repeat. (A) Deletions and insertions within the (GACC)_7_ΔT repeat that lead to Ade^+^ can be distinguished by their color. *ade6-(GACC)_7_*Δ*T* mutants form red colonies on medium with limited amount of adenine due to a defective *ade6* gene. They can revert to Ade^+^ by deletion of two or five repeat units, producing white Ade^+^, or by insertion of one or four repeat units, producing pink Ade^+^. (B) Percentage of deletions in the various strain backgrounds. Wild type, *msh3*, and *exo1* mainly reverted to Ade^+^ by deletions, while *msh2*, *msh6*, and *mlh1* mutants mainly reverted by insertions. Significantly different to wild type: ** p < 0.01; *** p < 0.001. Shown are average values with SDs. Reversion spectra are also presented as pie charts, with the proportion of deletions and insertions indicated in white and pink, respectively.

The nature of mutations was determined by sequencing of PCR products from genomic DNA using primers ade6_F 5′-ATTAACACTGATGCCTTGGC and ade6_R 5′-ACAGAGAACGTTTAGCGATC. In the case of *ade6-(GACC)_7_*Δ*T*, repeat tract changes were also analyzed by inspection of the color of Ade^+^ revertants (Figure 2A). The color was best determined when revertants were restreaked on YEA without supplemented adenine. The proportion of white and pink revertants was determined after 1–2 d of growth at 30°. Final averages and SDs were calculated from averages of at least three independent fluctuation tests, each with seven cultures, and up to 20 random revertants (where available) per culture.

### Data availability

*S. pombe* strains are available on request. The authors state that all data necessary for confirming the conclusions presented in the article are represented fully within the article.

## Results

### Genetic assay for microsatellite instability in S. pombe

We have previously reported that in-frame nucleotide insertions and codon changes in a region around nucleotide 1397 of the *ade6* gene [the ATG start codon is at 875 as defined by Szankasi *et al.* (1988)] in *S. pombe* did not disrupt or only slightly disrupted its functionality (Mansour *et al.* 2001; Marti *et al.* 2003). Here, we inserted tetra-, penta-, and hepta-nucleotide repeats in this part of *ade6* in order to analyze microsatellite instability in wild type and MMR mutants (Figure 1). Insertion of the repeats caused frameshifts, which rendered cells auxotrophic for adenine due to nearby located stop codons. Such *ade6* mutants required a supply of adenine for growth and turned red on the medium with a limited amount of adenine due to accumulation of a red pigment. Reversions of such strains to Ade^+^ can occur by deletions or insertions of repeats, when these events restore the open reading frame. With (GACC)_7_ΔT, (CTGCC)_6_, and (ATCGTCC)_7_, insertions of one repeat unit and deletions of two repeat units are the major events detectable. The opposite is the case with (GACC)_8_ and (ATCGTCC)_7_ΔT, where deletions of one repeat unit and insertions of two repeat units are the principal events that can be detected, although in the case of (ATCGTCC)_7_ΔT, we identified exclusive deletions as described below.

In the case *of ade6-(GACC)_7_*Δ*T*, deletions and insertion can be distinguished by the color of revertants (Figure 2A). Deletions caused white Ade^+^, while insertions caused pink Ade^+^. The pink color likely reflects that *ade6* is not fully functional and thus that the red pigment is produced in low quantities. Sequencing of 18 Ade^+^ revertants of the *(GACC)_7_*Δ*T* repeat in the various genetic backgrounds revealed deletions and sequencing of 23 *(GACC)_7_*Δ*T* revertants revealed insertions (Table 1). All revertants with deletions were white and all revertants with insertions were pink. Thus, the occurrence of deletions and insertions in the *(GACC)_7_*Δ*T* repeat can be easily determined from a large number of revertants. Analyzing revertants of the other *ade6* repeats did not allow a distinction by color, probably because Ade^+^ originating from deletions and insertions were both not fully functional. In these cases, PCR products of independent Ade^+^ revertants were subjected to sequencing to identify the number of repeats.

**Table 1:** Reversion spectra of tetra-nucleotide repeats

	(GACC)_8_						(GACC)_7_ΔT				
Relevant Genotype	(GACC)_7_ −4 bp	(GACC)_10_ +8 bp	(GACC)_13_ +20 bp	χ^2^ (p-Value)*^a^*			(GACC)_2_ΔT −20 bp	(GACC)_5_ΔT −8 bp	(GACC)_8_ΔT +4 bp	(GACC)_11_ΔT +16 bp	χ^2^ (p-Value) *vs.* WT
				*vs.* WT	*vs.* *msh2*	*vs.* *msh6*					
Wild type	0	10	0				0	7	2	1	
*msh2*	8	2	0	13.3 (2.6 × 10^−4^)			0	2	7	1	5.05 (0.025)
*msh3*	4	6	1	4.5 (0.034)	4.1 (0.044)	8.8 (0.03)	1	7	1	2	0.019 (0.89)
*msh6*	9	0	0	19 (1.3 × 10^−5^)			0	1	9	0	7.5 (0.0062)

Repeat tract changes were determined from random independent revertants. WT, wild type.

a χ^2^ and p-values (in parenthesis) are shown for the distribution of deletions *vs.* insertions and were calculated with an online program (http://www.socscistatistics.com/tests/chisquare/).

### GACC tetra-nucleotide repeats were unstable in msh2, msh6, and mlh1 mutants

We first analyzed stability of GACC tetra-nucleotide repeats in wild type, and in mutants deleted for either *msh2*, *msh3*, *msh6*, *mlh1*, or *exo1*. In wild type, *ade6-(GACC)_8_* and *ade6-(GACC)_7_*Δ*T* reverted to Ade^+^ with 1.2 × 10^−5^ and 2.1 × 10^−5^ reversions per cell division, respectively (Table 2). The *ade6-(GACC)_8_* reversion rates increased 39–44-fold in *msh2*, *msh6*, and *mlh1* mutants and slightly decreased in *msh3* and *exo1* mutants. Similarly, *ade6-(GACC)_7_*Δ*T* reversion rates increased 17–22-fold in *msh2*, *msh6*, and *mlh1* mutants and decreased 3–5-fold in *msh3* and *exo1* mutants. None of the differences between *msh3*, *exo1*, and wild type was statistically significant.

**Table 2:** Reversion rates of GACC tetra-nucleotide repeats

	Tetra-Nucleotide Repeat					
	(GACC)_8_			(GACC)_7_ΔT		
Relevant Genotype	Rate*^a^*	Fold Increase*^b^*	p-Value*^c^*	Rate	Fold Increase	p-Value
Wild type	1.2 ± 0.6 × 10^−5^	1		2.1 ± 2.0 × 10^−5^	1	
*msh2*	5.3 ± 2.7 × 10^−4^	44	0.002	4.6 ± 3.2 × 10^−4^	22	0.009
*msh3*	6.3 ± 3.6 × 10^−6^	0.5	0.15	5.2 ± 1.3 × 10^−6^	0.2	0.15
*msh6*	4.7 ± 2.8 × 10^−4^	39	0.006	4.6 ± 1.8 × 10^−4^	22	0.0001
*mlh1*	5.3 ± 2.5 × 10^−4^	44	0.002	3.5 ± 2.2 × 10^−4^	17	0.006
*exo1*	8.1 ± 0.9 × 10^−6^	0.7	0.33	7.3 ± 1.8 × 10^−6^	0.3	0.27

a Numbers are mean values with SDs.

b Relative to wild type.

c p-values were calculated by a two-tailed Student’s *t*-test in comparison to wild type.

Repeat tract changes in *ade6-(GACC)_7_*Δ*T* can be determined by colony color as described above and in *Materials and Methods*, and as illustrated in Figure 2A. In wild type, *msh3*, and *exo1* strains, reversions mainly occurred by deletions (Figure 2B). Most were deletions of two repeat units (Table 1). In contrast, *msh2*, *msh6*, and *mlh1* mutants mainly reverted to Ade^+^ by insertion of one repeat (Figure 2B and Table 1). Sequencing of Ade^+^ revertants of *ade6-(GACC)_8_* revealed that in wild type, all 10 had two repeat insertions (Table 1). In contrast, *msh2* and *msh6* reverted mainly by deletion of one repeat. The spectrum for *msh3* was more heterogeneous, with some preference for insertions. Importantly, the occurrence of deletions *vs.* insertions was significantly different to wild type (Table 1).

### Increased penta-nucleotide repeat stability in the msh3 mutant

The (CTGCC)_6_ repeat reverted in wild type with a rate of 3.4 × 10^−5^ to Ade^+^ (Table 3). Rates were not significantly different in *msh2*, *msh6*, *mlh1*, and *exo1* mutants. In contrast, the (CTGCC)_6_ repeat appeared to be more stable in the *msh3* mutant. It reverted mostly through gain of one repeat unit in all strain backgrounds, with the possible exception of *mlh1* (Table 4). We conclude that MutSα cannot repair loops with five unpaired nucleotides in the (CTGCC)_6_ context, and that Msh3 has some function in stability of the penta-nucleotide repeat.

**Table 3:** Reversion rates of the (CTGCC)_6_ repeat

Relevant Genotype	Penta-Nucleotide Repeat (CTGCC)_6_		
	Rate*^a^*	Fold Increase*^b^*	p-Value*^c^*
Wild type	3.4 ± 0.5 × 10^−5^	1	
*msh2*	5.5 ± 1.9 × 10^−5^	1.6	0.08
*msh3*	1.5 ± 0.2 × 10^−5^	0.4	0.0005
*msh6*	4.9 ± 2.7 × 10^−5^	1.4	0.32
*mlh1*	4.3 ± 0.7 × 10^−5^	1.3	0.09
*exo1*	3.5 ± 1.2 × 10^−5^	1	0.95

a Numbers are mean values with SDs.

b Relative to wild type.

c p-values were calculated by a two-tailed Student’s *t*-test in comparison to wild type.

**Table 4:** Reversion spectra of the (CTGCC)_6_ repeat

	(CTGCC)_6_		
Relevant Genotype	(CTGCC)_4_ −10 bp	(CTGCC)_7_ +5 bp	(CTGCC)_10_ +20 bp
Wild type	1	15	0
*msh2*	1	9	0
*msh3*	0	10	0
*msh6*	2	7	1
*mlh1**^a^*	6	10	0
*exo1*	2	8	0

Repeat tract changes were determined from random independent revertants.

a Distribution of deletions and insertions in *mlh1* background significantly different to wild type (χ^2^ = 4.57; p = 0.033). Reversion spectra of all other mutants were not significantly different to wild type.

### Hepta-nucleotide repeat stability was not affected in msh2, msh6, and mlh1 and was slightly decreased in msh3 and exo1 mutants

In wild type, the hepta-nucleotide repeats *ade6-(ATCGTCC)_5_*Δ*T* and *ade6-(ATCGTCC)_5_* reverted to Ade^+^ at rates of 7.2 × 10^−6^ and 7.5 × 10^−6^, respectively (Table 5). Rates were not significantly different in *msh2*, *msh6*, and *mlh1* mutants, suggesting that loops with seven unpaired nucleotides are not repaired by MMR in *S. pombe*. In *msh3* and *exo1* mutants, reversion rates were increased 1.7–1.9-fold for *ade6-(ATCGTCC)_5_*Δ*T* (not significant) and 2.8–2.9-fold for *ade6-(ATCGTCC)_5_* (significant) (Table 5). All sequenced *ade6-(ATCGTCC)_5_*Δ*T* revertants contained four hepta-nucleotide repeats and thus originated from deletion of one repeat (Table 6). The fact that insertions were not found at all could be either due to a relative low sample size or that insertion of 14 nucleotides, leading to a total of 39 additional nucleotides in *ade6* (Figure 1), does not render cells Ade^+^. *ade6-(ATCGTCC)_5_* reverted mainly by insertion of one repeat and less frequently by deletion of two repeats without any significant differences between the spectra of wild type and any of the mutants (Table 6).

**Table 5:** Reversion rates of hepta-nucleotide repeats

	Hepta-Nucleotide Repeat					
	(ATCGTCC)_5_ΔT			(ATCGTCC)_5_		
Relevant Genotype	Rate*^a^*	Fold Increase*^b^*	p-Value*^c^*	Rate	Fold Increase	p-Value
Wild type	7.2 ± 3.2 × 10^−6^	1		7.5 ± 2.9 × 10^−6^	1	
*msh2*	7.3 ± 1.8 × 10^−6^	1	0.99	6.8 ± 0.9 × 10^−6^	0.9	0.7
*msh3*	1.4 ± 0.7 × 10^−5^	1.9	0.13	2.1 ± 0.6 × 10^−5^	2.8	0.0008
*msh6*	5.2 ± 3.2 × 10^−6^	0.7	0.4	1.0 ± 0.4 × 10^−5^	1.3	0.34
*mlh1*	6.2 ± 2.3 × 10^−6^	0.9	0.66	4.7 ± 0.9 × 10^−6^	0.6	0.15
*exo1*	1.2 ± 0.4 × 10^−5^	1.7	0.12	2.2 ± 0.9 × 10^−5^	2.9	0.0066

a Numbers are mean values with SDs.

b Relative to wild type.

c p-values were calculated by a two-tailed Student’s *t*-test in comparison to wild type.

**Table 6:** Reversion spectra of hepta-nucleotide repeats

	(ATCGTCC)_5_ΔT			(ATCGTCC)_5_	
Relevant Genotype	(ATCGTCC)_4_ΔT −7 bp	(ATCGTCC)_7_ΔT +14 bp		(ATCGTCC)_3_ −14 bp	(ATCGTCC)_6_ +7 bp
Wild type	5	0		3	8
*msh2*	5	0		4	10
*msh3*	5	0		4	7
*msh6*	5	0		2	12
*mlh1*	5	0		3	10
*exo1*	5	0		2	11
*rad2*	ND	ND		6	6
*rad50*	ND	ND		3	9
*rad51*	ND	ND		7	13

Repeat tract changes were determined from randomly selected revertants of independent cultures. None of the reversion spectra of the mutants is significantly different to wild type. ND, not determined.

### Hepta-nucleotide repeat stability in rad2^FEN1^, rad50, and rad51 mutants

The *msh3* and *exo1* mutants, but not the mutants *msh2*, *msh6*, and *mlh1*, exhibited increased instability of the (ATCGTCC)_5_ repeat (Table 5). Since defective *msh2* or *mlh1* is generally considered to completely inactivate MMR, the (ATCGTCC)_5_ repeat instability in *msh3* and *exo1* is not due to a defect in MMR. We therefore wanted to analyze the genetic context of *msh3* and *exo1* defects in microsatellite stability. To do this, we measured reversion rates of the hepta-nucleotide repeat in *rad2^FEN1^*, *rad50*, and *rad51* mutants and in various double mutants (Table 7). FEN1 and HR have been implicated in repeat stability in *S. cerevisiae* (Johnson *et al.* 1995; Tishkoff *et al.* 1997; Freudenreich *et al.* 1998; Kirchner *et al.* 2000; Sundararajan *et al.* 2010). We found that the *rad2^FEN1^*, *rad50*, and *rad51* single mutants had ∼2-fold increased reversion rates (Table 7). The *msh3 exo1* double mutant reverted significantly less frequently to Ade^+^ than either single mutant. Mutation rates remained about the same in *msh3 rad50*, *msh3 rad2^FEN1^*, and *exo1 rad50* but decreased in the *msh3 rad51* and *exo1 rad51* double mutants when compared to respective single mutants (Table 7). Like wild type and the other single mutants, *rad50* and *rad51* strains mainly reverted to Ade^+^ by insertion of one repeat unit (Table 6). In the *rad2^FEN1^* mutant, 50% of the reversions were due to deletion of two repeats, which was not significantly different to wild type. A reduction of reversion rates in the *msh3 rad51* and *exo1 rad51* double mutants indicates that Exo1 and Rad51 act error prone on the (ATCGTCC)_5_ repeat when *msh3* is mutated.

**Table 7:** Reversion rates of (ATCGTCC)_5_ repeats in *msh3*, *exo1*, and *rad* mutants

	(ATCGTCC)_5_					
			p-Values*^c^*			
Relevant Genotype	Rate*^a^*	Fold Increase*^b^*	*vs.* WT	*vs.* *msh3*	*vs.* *exo1*	*vs.* *rad*
Wild type	7.5 ± 2.9 × 10^−6^	1				
*msh3*	2.1 ± 0.6 × 10^−5^	2.8	0.0008			
*exo1*	2.2 ± 0.9 × 10^−5^	2.9	0.0066			
*msh3 exo1*	5.7 ± 0.5 × 10^−6^	0.8		0.0049	0.032	
*rad2*	1.5 ± 0.7 × 10^−5^	2	0.043			
*rad50*	1.4 ± 0.4 × 10^−5^	1.9	0.016			
*rad51*	1.7 ± 0.6 × 10^−5^	2.3	0.0087			
*msh3 rad2*	2.1 ± 0.5 × 10^−5^	2.8		0.98		0.24
*msh3 rad50*	2.6 ± 0.7 × 10^−5^	3.5		0.26		0.033
*msh3 rad51*	2.2 ± 2.1 × 10^−6^	0.3		0.0018		0.01
*exo1 rad50*	2.7 ± 0.8 × 10^−5^	3.6			0.24	0.025
*exo1 rad51*	7.0 ± 3.1 × 10^−6^	0.9			0.011	0.013

a Numbers are mean values with SDs. Values from wild type, *msh3*, and *exo1* derived from Table 5 and are shown for comparison.

b Relative to wild type.

c p-values were calculated by a two-tailed Student’s *t*-test in comparison to the indicated strains. WT, wild type; *rad*, *rad2^FEN1^*, *rad50*, or *rad51* single mutants.

## Discussion

### MutSα and MutLα are essential for S. pombe MMR, which is limited to loops with up to four nucleotides

Eukaryotic MMR is initiated by MutSα for repair of base-base mismatches and loops or by MutSβ for repair of loops (Marti *et al.* 2002; Jiricny 2013). In *S. cerevisiae*, *msh3* and *msh6* mutants show little to moderate increases of mutation rates in mono- and dinucleotide repeats (Johnson *et al.* 1996; Marsischky *et al.* 1996; Greene and Jinks-Robertson 1997; Sia *et al.* 1997). On the other hand, such repeats are highly unstable in *msh3 msh6* double mutants and within the range of the *msh2* instability, indicating redundancy of MutSα and MutSβ for small loops in this organism. In humans, MutSα is the major factor for recognition of base-base mismatches and loops, while MutSβ rather serves as a backup (Drummond *et al.* 1997; Genschel *et al.* 1998; Marra *et al.* 1998).

In *S. pombe*, we knew to this date that MMR is able to repair base-base mismatches and loops with up to two nucleotides (Schär *et al.* 1997; Rudolph *et al.* 1999; Mansour *et al.* 2001; Tornier *et al.* 2001; Marti *et al.* 2003). This requires MutSα and MutLα but not MutSβ. In the present study, we expanded analysis of loop repair in *S. pombe* to four to seven unpaired nucleotides. Our aim was to determine the contributions of Msh2, Msh3, Msh6, Mlh1, and Exo1, and particularly the relative roles of MutSα and MutSβ in stability of repeats with four or more iterated nucleotides in this model organism. The microsatellites tested were (GACC)_7_ and (GACC)_8_ tetra-, (CTGCC)_6_ penta-, and (ATCGTCC)_5_ hepta-nucleotide repeats (Figure 1). All such insertions caused a frameshift, rendering cells defective in *ade6*. Reversions to Ade^+^ occurred when deletions or insertions of repeats restored the open reading frame.

Inactivated Msh2, Msh6, and Mlh1 caused instability of the tetra-nucleotide repeats, while defective Msh3 and Exo1 rather made the repeats slightly more stable, although this was not significantly different to wild type (Table 2). The (GACC)_8_ repeat reverted in wild type by insertions of eight nucleotides, while in *msh2* and *msh6* mutants, mainly deletions of four nucleotides occurred (Table 1). Thus, MutSα of *S. pombe* is capable to initiate MMR of loops with four unpaired nucleotides, whereas MutSβ is not.

The assay with the (GACC)_7_ΔT repeat allowed distinguishing deletions from insertions by the color of Ade^+^ revertants (Figure 2A). We found that wild type, *msh3*, and *exo1* mainly reverted by eight-nucleotide deletions, and *msh2*, *msh6*, and *mlh1* mainly by four-nucleotide insertions (Figure 2B and Table 1). Thus, this assay also revealed that MutSα but not MutSβ initiates MMR of loops with four nucleotides. In addition, Mlh1, and by extrapolation MutLα, is involved in removal of four-nucleotide loops. Since four-nucleotide deletions, detectable with (GACC)_8_, and four-nucleotide insertions, detectable with (GACC)_7_ΔT, were the predominant reversion events in *msh2* and *msh6* mutants, slippage of one repeat can occur in the template and in the nascent strand during replication, and both types of events are corrected by MMR mediated by MutSα and MutLα.

Mutation rates of the penta-nucleotide repeat (CTGCC)_6_ in *msh2*, *msh6*, *mlh1*, and *exo1* mutants were similar to that of wild type, but decreased in *msh3* (Table 3). All strains preferentially reverted to Ade^+^ by insertion of one repeat (Table 4). Inactivation of *msh2*, *msh6*, and *mlh1* did not affect reversion rates or spectra of the hepta-nucleotide repeats (ATCGTCC)_5_ΔT and (ATCGTCC)_5_ (Table 5 and Table 6). Like wild type, the mutants reverted by deletion of one repeat in (ATCGTCC)_5_ΔT and mainly by insertion of one repeat in (ATCGTCC)_5_. We conclude that loops in penta- and hepta-nucleotide repeats are not substrates of MutSα and MutLα in *S. pombe*.

### Msh3 has an MMR-independent function in repeat stability

Our previous data showed that *msh3* mutants had no significant defects in repair of base-base mismatches and of loops with one unpaired nucleotide in a T_6_ repeat and in nonrepetitive DNA (Tornier *et al.* 2001; Marti *et al.* 2003). *msh3* mutations caused some instability of a (GT)_8_ dinucleotide repeat, which was mostly evident by a reversion spectrum different to wild type (Mansour *et al.* 2001). Wild type reverted mainly by four-nucleotide insertions, whereas *msh3* mainly reverted by two-nucleotide deletions in the (GT)_8_ repeat. However, this was clearly less frequent than in *msh2*, *msh6*, and *pms1* mutants (Mansour *et al.* 2001). In the present study, we also found that the spectrum for the (GACC)_8_ repeat was different to wild type and to *msh2* and *msh6* (Table 1). Wild type exclusively reverted via eight-nucleotide insertions (10 out of 10 revertants analyzed), and *msh2* and *msh6* mostly by four-nucleotide deletions. In contrast, four out of 11 revertants in *msh3* background were due to four-nucleotide deletions, six originated from eight-nucleotide insertions, and one by an insertion of 20 nucleotides (Table 1). Thus, Msh3 appears to have a function in tetra-nucleotide repeat stability, which is different to Msh2 and Msh6.

In the case of the penta-nucleotide repeat, we found that the *msh3* mutant showed a lower mutation rate than wild type (Table 3), indicating a role of Msh3 in supporting tract length changes in this repeat. Intriguingly, the *msh3* mutant exhibited increased instability of the hepta-nucleotide repeats (Table 5). Thus, Msh3 has a function in maintaining stability of such repeats. Since *msh2*, *msh6*, and *mlh1* did not show instability of hepta-nucleotide repeats, the Msh3 function appears to be MMR independent. We have found a genetic link to Rad50, Rad51, and Exo1, indicating that the Msh3 function is related to recombinational processes. Such a function is well known for *S. cerevisiae* MutSβ, which participates in SSA where repeats flank a double-strand break (Sugawara *et al.* 1997; Chakraborty and Alani 2016). MutSβ of *S. pombe* likely acts similarly, as both Msh2 and Msh3 have a function in the recombinational process of mating-type switching (Fleck *et al.* 1992; Rudolph *et al.* 1999). However, genetic data presented here and in our previous studies suggest that the functions of Msh3 in repeat stability and recombination is likely independent of Msh2 (Tornier *et al.* 2001; Mansour *et al.* 2001; Marti *et al.* 2003). The Msh2 independent role of *S. pombe* Msh3 in recombination may relate to that of bacterial MutS2 proteins, which act in recombination and antirecombination mechanisms but not in MMR (Pinto *et al.* 2005; Burby and Simmons 2017). MutS2 of *Helicobacter pylori* binds to DNA structures that resemble recombination intermediates and inhibits strand exchange *in vitro* (Pinto *et al.* 2005). In this regard, *S. pombe* Msh3 may be functionally similar, although structurally, it lacks the endonuclease domain of MutS2, and homology of its amino acid sequence clearly allocates it to the group of eukaryotic Msh3 proteins.

Structural studies with human MutS heterodimers showed that mismatch binding largely occurs by Msh3 or Msh6, while Msh2 has few contacts with the DNA backbone of correctly paired nucleotides in the vicinity (Warren *et al.* 2007; Gupta *et al.* 2011). The human Msh6 protein interacts directly with mismatched bases via a phenylalanine, which is conserved in eukaryotic Msh6 orthologs and bacterial MutS. In contrast, human Msh3 lacks this residue and instead interacts with phosphate groups of the unpaired nucleotides (Gupta *et al.* 2011). Work by Lee *et al.* (2007) demonstrated that deletion of the mismatch binding domain of *S. cerevisiae* Msh2 causes loss of MutSβ−dependent MMR activity and revealed that the domain in Msh2 is required for general DNA binding, and in Msh3 for binding to DNA loops. In complex with Msh2, a chimeric Msh6 protein of *S. cerevisiae* containing the mismatch binding domain of Msh3 showed substrate specificity of Msh3, *i.e.*, high affinity to loops with one to four unpaired nucleotides (Shell *et al.* 2007). The amino acid sequence within the mismatch binding domain of *S. pombe* Msh3 is very similar to that of human Msh3. However, in contrast to *S. cerevisiae* and human Msh3 and orthologs of other eukaryotes, *S. pombe* Msh3 lacks a canonical PIP box, which mediates interaction with PCNA. The PIP box of human Msh3 overlaps with the Mlh1 binding domain (Iyer *et al.* 2010). Thus, it is also conceivable that amino acid residues required for interaction with Mlh1 are not present in *S. pombe* Msh3. It is currently not known whether *S. pombe* Msh3 can interact with PCNA or MutLα. If it does not, this may explain that it does not participate in MMR.

### Role of MutSα and MutSβ in MMR

The *Escherichia coli* homodimer MutS enables repair of base-base mismatches and loops with up to four nucleotides (Iyer *et al.* 2006). Reconstituted MMR with *S. cerevisiae* proteins *in vitro* revealed that both MutSα and MutSβ could initiate repair of base-base mismatches and of loops with one, two, or four nucleotides (Bowen *et al.* 2013). Human MutSα binds to base-base mismatches and to loops with up to eight nucleotides, while MutSβ allows repair of loops with two to about eight nucleotides (Genschel *et al.* 1998). Our genetic data imply that *S. pombe* Msh6 as part of the MutSα heterodimer is able to bind to loops with up to four unpaired nucleotides. Thus, the substrate spectrum of bacterial MutS and MutSα of *S. pombe* appears to be similar. During evolution, the spectrum had been extended to enable recognition of larger loops in humans. On the other hand, the substrate spectrum of MutSβ considerably differs between species. Human MutSβ supports repair of loops with two to eight nucleotides (Genschel *et al.* 1998), whereas *S. cerevisiae* MutSβ is also involved in repair of some base-base mismatches besides loop repair (Harrington and Kolodner 2007), and *S. pombe* MutSβ apparently does not have a function in MMR. In addition, some eukaryotes, such as *Caenorhabditis elegans* and *Drosophila melanogaster* do not have an Msh3 ortholog (Marti *et al.* 2002) and likely carry out MMR with MutSα and MutLα and no other MutS and MutL heterodimers, like *S. pombe* does.

Harrington and Kolodner (2007) interpreted mutation spectra of base substitutions in *S. cerevisiae msh3* mutants that were different to wild type as a role of MutSβ in MMR of base-base mismatches. We observed differences of *msh3* in mutation spectra for a (GT)_8_ repeat (Mansour *et al.* 2001), a reduction of recombination events (Tornier *et al.* 2001), and of reversion rates at the (CTGCC)_6_ repeat (Table 3), an altered reversion spectrum for (GACC)_8_ (Table 1) and repeat instability of (ATCGTCC)_5_ (Table 5). We interpret these differences as phenotypes caused by loss of MMR-independent functions of Msh3.

### Does S. pombe Exo1 have a function in MMR?

Exo1 of *S. pombe* was the first eukaryotic exonuclease to be identified as having a function in repair of mismatches (Szankasi and Smith 1995). Further studies with *S. pombe* showed that Exo1 contributes to MMR of base-base mismatches (Rudolph *et al.* 1998), modulates MMR of two-nucleotide loops in nonrepetitive DNA (Marti *et al.* 2003), and has an MMR-independent function in dinucleotide repeat stability (Mansour *et al.* 2001). In the present study, we did not found any evidence for a role of Exo1 in tetra- and penta-nucleotide repeat stability (Table 2 and Table 3). However, we observed that loss of Exo1 caused instability of hepta-nucleotide repeats, in contrast to the MMR mutants *msh2*, *msh6*, and *mlh1* (Table 5). Exo1 also acts in recombination and double-strand break repair (Fiorentini *et al.* 1997; Tsubouchi and Ogawa 2000; Kirkpatrick *et al.* 2000; Cejka 2015). Thus, a defect in a recombination mechanism might cause hepta-nucleotide instability of the *S. pombe exo1* mutant rather than MMR deficiency, as discussed below.

Although a nuclease is essential for removal of unpaired nucleotides during MMR, Exo1 does seem to be dispensable for MMR-mediated loop repair in *S. pombe*. This may be attributed to redundancy with other nucleases. MutLα of *S. cerevisiae* and humans has endonuclease activity, which is sufficient for completing MMR in the absence of Exo1 (Kadyrov *et al.* 2006, 2007; Smith *et al.* 2013; Goellner *et al.* 2014, 2015). Thus, it is also likely that Exo1 of *S. pombe* participates in MMR, but that MutLα and maybe other nucleases can replace its function. In fact, the amino acids required for MutLα nuclease activity are all highly conserved between eukaryotes, including *S. pombe* (Smith *et al.* 2013), supporting the idea that having endonuclease activity is a general feature of eukaryotic MutLα. In *S. cerevisiae*, *exo1* mutants exhibit weak defects in MMR (Tishkoff *et al.* 1997; Amin *et al.* 2001; Smith *et al.* 2013; Goellner *et al.* 2014), likely because MutLα and Exo1 nuclease activities are largely redundant. *exo1* deletion strains and *pms1* strains with mutations causing endonuclease deficiency generally showed subtle increases of mutation rates, which strongly increased when both mutations were combined (Smith *et al.* 2013).

### Rad51 and Exo1 are involved in error prone repair at (ATCGTCC)_5_ repeats in msh3 mutants

Stability of the hepta-nucleotide repeat (ATCGTCC)_5_ was influenced by processes involving Msh3, Exo1, Rad2^FEN1^, Rad50, and Rad51. Deletions of any of the genes caused an ∼2–3-fold increase of reversion rates, which was predominantly due to expansions by one repeat unit and therefore by insertions in the nascent strand (Table 6 and Table 7). Rates were not further increased in the *msh3 rad2^FEN1^*, *msh3 rad50*, and *exo1 rad50* double mutants. Instead, the *msh3 exo1*, *msh3 rad51*, and *exo1 rad51* double mutants had lower rates than the respective single mutants. In *S. cerevisiae*, CAG trinucleotide repeats were unstable in *rad51*, *rad52*, and *mre11* single mutants (Sundararajan *et al.* 2010). However, increased rates of repeat expansions in *mre11* were largely suppressed by additional mutation of *rad52*. These data suggest that the MRN complex plays a role in maintaining repeat stability, and that downstream steps of HR in *mre11*, but not in wild-type background, can carry out error prone recombination at repeats (Sundararajan *et al.* 2010). In summary, the (ATCGTCC)_5_ repeat analyzed in our study might be stabilized by Msh3 and slipped-out loops correctly processed by HR requiring Rad50, Exo1, and Rad51, thereby preventing aberrant events. In the absence of Msh3, the Exo1 and Rad51 proteins might carry out error prone processes, such as misalignment of repeats after strand resection catalyzed by Exo1 and during strand invasion mediated by Rad51.

### Conclusions

We conclude from our studies that *S. pombe* Msh6, as part of MutSα, recognizes base-base mismatches and loops with one to four unpaired nucleotides, while Msh3 does not play a significant role in MMR, but rather maintains repeat stability independently of MMR. Consequently, *S. pombe* MMR cannot repair loops with five or more nucleotides, in contrast to human MMR (Genschel *et al.* 1998). Microsatellites with five or six iterated nucleotides are rare in *S. pombe* (hepta-nucleotide repeats were not analyzed) (Karaoglu *et al.* 2005), but are relatively abundant in the human genome (Lander *et al.* 2001). Thus, to ensure genome stability, humans require repair of larger loops that occur by strand slippage in microsatellites, while larger loops may be formed rarely in *S. pombe* microsatellites. It is therefore critical for humans, but not for *S. pombe*, to have an MMR system that can deal with larger loops.
